# Radiation Segmentectomy for Primary and Metastatic Liver Tumors: Current Evidence, Tumor-Specific Dosimetric Rationale, and the Need for Standardization

**DOI:** 10.3390/cancers18213501

**Published:** 2026-10-30

**Authors:** Dimitrios Xenos, Vlasios S. Sotirchos, Bruno Solis, Platon M. Dimopoulos, Achilles Chatziioannou, Miltos Krokidis, Constantinos T. Sofocleous

**Affiliations:** 1 Interventional Radiology Service, Memorial Sloan Kettering Cancer Center, New York, NY 10065, USA; 2 Department of Interventional Radiology, University Hospital of Patras, 26504 Patras, Greece; 3 1st Department of Radiology, School of Medicine, Areteion Hospital, National and Kapodistrian University of Athens, 11528 Athens, Greece

**Keywords:** radiation segmentectomy, yttrium-90, radioembolization, hepatocellular carcinoma, intrahepatic cholangiocarcinoma, liver metastases, dosimetry, tumor vascularity

## Abstract

**Background**: Radiation segmentectomy (RS) is the selective trans-arterial delivery of an ablative dose of radiation to tumor-bearing hepatic segments, with the goal of complete tumor necrosis comparable to resection or thermal ablation. The technique was first described in patients with unresectable hepatocellular carcinoma (HCC). RS has since been applied to intrahepatic cholangiocarcinoma (iCCA) and to liver metastases of diverse origin, including colorectal, neuroendocrine, breast and sarcoma primaries. **Purpose**: To synthesize the original-data evidence base for RS across tumor types, to demonstrate the impact of tumor vascularity on tumor-specific dosimetry methods, and to define where the evidence supports standardization of methodology. **Findings**: Evidence is strongest for HCC, where pathologic-explant studies tie ablative dose thresholds to complete pathologic necrosis and prospective and large-cohort data demonstrate high response rates and extended patient survival. For colorectal liver metastases, several studies have associated tumor absorbed dose with response and local control. A single study additionally suggests that the dose delivered to the peritumoral margin is associated with local tumor control, an interesting hypothesis that requires further prospective validation. Data in iCCA and neuroendocrine metastases support feasibility and durable responses. The first international dosimetry consensus for non-HCC indications appeared in 2025. Heterogeneity in dosimetry models, dose thresholds, patient selection, and outcome reporting limits cross-study pooling. **Conclusions**: RS serves as a potentially curative locoregional therapy for selected patients with early-stage HCC and represents a promising treatment option for non-HCC tumors, especially for those not amenable to resection or thermal ablation. Histology-specific dosimetry, prospective validation, especially in non-HCC disease, and consensus reporting standards are necessary.

## Simple Summary

Radiation segmentectomy is a procedure designed to target liver segments containing tumors by administering a high dose of radiation trans-arterially, with the objective of achieving complete tumor destruction. Initially developed for patients with hepatocellular carcinoma (HCC) that could not be surgically excised, it has since been expanded to treat intrahepatic cholangiocarcinoma and liver metastases originating from various cancers. This review consolidates the original data on radiation segmentectomy across different tumor types, emphasizing the influence of tumor vascularity on tumor-specific dosimetry techniques and identifying areas where evidence supports methodological standardization. Variability in dosimetry models, dose thresholds, patient selection, and outcome reporting impedes the ability to aggregate data across studies. Radiation segmentectomy represents a potentially curative locoregional therapy for selected patients with early-stage HCC and demonstrates potential efficacy for non-HCC tumors. There is a need for histology-specific dosimetry, prospective validation, particularly in non-HCC diseases, and standardized reporting practices.

## 1. Introduction

Radiation segmentectomy (RS) delivers an ablative dose of radiation within hepatic segments containing tumor while sparing non-involved parenchyma. The original 84-patient hepatocellular carcinoma (HCC) series established that segmental delivery could achieve a median segmental dose of 521 Gy and an estimated tumor dose of 1214 Gy, with 81% tumor necrosis [European Association for the Study of the Liver (EASL) necrosis criteria], 59% imaging response [World Health Organization (WHO) size criteria], a median time to progression of 13.6 months, and a median survival of 26.9 months [1]. The biological premise is that the short mean tissue penetration of the ^90^Y beta particle activity confines high-dose radiation to the targeted angiosome.

The central theme of this review is that tumor vascular architecture and properties, not tumor type per se, govern microsphere deposition and distribution. Thus, tumor vascularity needs to be considered in dosimetry planning. Hypervascular tumors, such as HCC and neuroendocrine metastases, support tumor-volume based dosimetry, whereas in the hypovascular colorectal metastases, the most relevant target may be concentrated in the peritumoral rim, a hypothesis that requires further prospective validation. In this work, we deliberately separated what the data have established from what remains biologically plausible but not yet proven.

### Literature Search and Selection

PubMed, Embase and Scopus were searched from January 1995 to 30 September 2026 using the following search algorithms:

PubMed: (“radiation segmentectomy”[tiab] OR “segmental radioembolization”[tiab] OR “segmental radioembolisation”[tiab] OR radioembolization[tiab] OR “selective internal radiation therapy”[tiab] OR “Yttrium Radioisotopes”[MeSH] OR “yttrium-90”[tiab] OR “^90^Y”[tiab] OR “Y90”[tiab]) AND (“Carcinoma, Hepatocellular”[MeSH] OR “hepatocellular carcinoma”[tiab] OR “Cholangiocarcinoma”[MeSH] OR cholangiocarcinoma[tiab] OR “Liver Neoplasms”[MeSH] OR “liver metastases”[tiab] OR “hepatic metastases”[tiab] OR colorectal[tiab] OR neuroendocrine[tiab]) AND (segment*[tiab] OR dosimetry[tiab]).

Embase: (‘radiation segmentectomy’:ti,ab OR ‘segmental radioembolization’:ti,ab OR radioembolization:ti,ab OR ‘selective internal radiation therapy’:ti,ab OR ‘yttrium 90’/exp) AND (‘liver cell carcinoma’/exp OR ‘cholangiocarcinoma’/exp OR ‘liver metastasis’/exp OR colorectal:ti,ab OR neuroendocrine:ti,ab) AND (segment*:ti,ab OR ‘dosimetry’/exp).

Scopus: ((“radiation segmentectomy” OR “segmental radioembolization” OR radioembolization OR “selective internal radiation therapy” OR “yttrium-90” OR ^90^Y) AND (“hepatocellular carcinoma” OR cholangiocarcinoma OR “liver metastases” OR “hepatic metastases” OR colorectal OR neuroendocrine) AND (segment* OR dosimetry)).

The reference lists of the retrieved articles were systematically examined. We included full-text, English-language original studies that addressed response, local control, survival, pathologic necrosis, dosimetry, or toxicity following radiation segmentectomy or segmental radioembolization. Series that combined segmental and lobar treatments were included only if the proportion of segmental treatment was specified; this proportion is detailed in the text and tables. Case reports and conference abstracts were excluded. Studies and recommendations not specific to segmental delivery are cited solely for context and are identified accordingly.

## 2. Technical and Dosimetric Foundations

### 2.1. Microsphere Products

Two yttrium-90 (^90^Y) microsphere products are used, glass and resin, and their dose thresholds are device-specific. In a glass-microsphere explant series, complete pathologic necrosis was achieved in 53% of tumors receiving > 190 Gy and in 75% of those receiving > 500 Gy by single-compartment Medical Internal Radiation Dosimetry (MIRD), indicating a dose-dependent necrosis relationship [2]. In a resin-microsphere series, a post-treatment positron emission tomography/computed tomography (PET/CT) minimum dose absorbed by 50% of tumor volume of at least 300 Gy was associated with a lower 2-year disease progression rate than doses below 300 Gy (17% vs. 61%, *p* = 0.047) [3]. When the two devices were compared head-to-head with a single voxel-based method, the objective response and disease control rates showed no significant difference between resin and glass (95% vs. 82%, *p* = 0.1; 100% vs. 95%, *p* = 0.1), although resin was delivered at markedly lower mean tumor doses (308 vs. 794 Gy, *p* = 0.0002) and achieved a significantly higher complete response rate (95% vs. 56%, *p* = 0.003); device-specific mean tumor dose thresholds for complete response were 247 Gy for resin and 481 Gy for glass [4].

### 2.2. Dosimetry Methods

Improvements in the safety and efficacy of ^90^Y radioembolization have paralleled a progressive refinement in dosimetry. Empirical whole-liver methods have increasingly been replaced by compartmentalized, tumor-specific dosimetry that improves target tumor precision and spares uninvolved parenchyma. The body-surface-area (BSA) method designed for whole-liver administration was never intended to calculate an individualized tumor dose [3,5]. The single-compartment MIRD model calculates prescribed activity based on the targeted parenchymal volume, accounting for lung-shunt fraction [3,6]. The partition model was designed to separate tumor, non-involved-liver parenchyma, and lung into compartments so that high tumor dose could be delivered while constraining non-involved tissue dose [7]. The DOSISPHERE-01 randomized trial showed that personalized, multicompartment dosimetry is superior to a standard approach in locally advanced HCC [8]. Voxel-based dosimetry from post-treatment imaging resolves the three-dimensional dose distribution and has been used to correlate the maximum tumor-absorbed dose with response [5] and to relate sphere concentration and the minimum dose covering 70%, 90%, and 99% of the tumor volume (D70, D90, D99) to complete pathologic necrosis [9]. Beyond total dose, microsphere specific activity is an independent determinant of necrosis: an explant analysis identified treatment intensification and specific activity as predictors of complete pathologic necrosis (CPN) [10], and a comprehensive glass-microsphere analysis found higher specific activity, tumor dose, and angiosome dose in tumors achieving CPN [11]. For non-HCC indications, an international working group recommends a mean-dose framework in which single-compartment dosimetry is acceptable for radiation segmentectomy and multicompartment dosimetry is preferred for evaluating tumor and normal-tissue absorbed doses [12].

A retrospective study reported no statistically significant differences in efficacy or safety without technetium-99m macroaggregated albumin (MAA) pre-treatment work-up in HCC within the Milan criteria. A retrospective study compared 38 patients who underwent standard trans-arterial radioembolization with MAA-based lung shunt estimation with 62 who underwent a streamlined protocol omitting it entirely. Complete response rates did not differ significantly (92.1% vs. 90.3%), with no significant difference in time to progression or adverse events, and, critically, no radiation pneumonitis in either group [13]. The safety of omitting MAA requires further validation in prospective studies.

### 2.3. Segmental Dose Versus Tumor Absorbed Dose

Reported absorbed doses are model-dependent and cannot be interpreted without specifying the dosimetry model used, since a single administered activity yields different calculated doses according to whether it is assigned to the perfused volume as one compartment or apportioned between tumor and non-tumoral parenchyma [14,15]. Under single-compartment MIRD, the historic standard for radiation segmentectomy, microspheres are assumed to distribute uniformly through the perfused volume, so the segmental (perfused-volume) dose and the tumor dose are identical by construction rather than by measurement, and a single value is reported [1,7]. This equivalence is stated explicitly in metastatic series, where the segment dose equals the tumor dose [16]. Where compartments are distinguished by partition modeling, the administered activity is first apportioned between tumor and non-tumoral liver according to their masses and relative uptake, and only then converted to compartmental doses [7]. Where the distribution is instead measured on post-treatment imaging, the tumor-to-non-tumor dose ratio has a median of 1.6 and ranges from 0.8 to 4.5, so a single perfused-volume value can understate or overstate the dose actually delivered to tumor [9]. An international dosimetry consensus for non-HCC indications endorsed single-compartment dosimetry at ≥400 Gy to the perfused volume for radiation segmentectomy in intrahepatic cholangiocarcinoma, colorectal liver metastases, and neuroendocrine liver metastases [12]. Consistent with that threshold, the median absorbed dose to the treated liver volume was 410.1 Gy (IQR 199.7–797.7) in the LEGACY study [17] and the median single-compartment dose across targeted angiosomes was 403 Gy (IQR 303–522) in a percent-liver-treated analysis [18].

Multicompartment and voxel-based methods resolve the tumor and the perfused parenchyma separately. In hypervascular HCC that concentrates microspheres, the tumor dose is roughly twice the segmental dose in paired reports (Table 1): 521 Gy segmental versus 1214 Gy tumor dose in the original description [1], 254 versus 536 Gy with superselective delivery [19], a median perfused-segment dose of 584 Gy versus a median tumoral dose of 1004.6 Gy in the prospective RASER cohort [20], and an angiosome dose of 577 Gy versus a tumor dose of 1086 Gy in tumors achieving complete pathologic necrosis [11]. In the same explant cohort, single-compartment dosimetry predicted complete pathologic necrosis at ≥439 Gy and multicompartment tumor dosimetry at ≥844 Gy, illustrating that the same ablative effect is described by different numbers depending on the dosimetry model, with similar predictive performance between the two methods when both were optimized per their respective thresholds [11].

## 3. Hepatocellular Carcinoma

### 3.1. Clinical Outcomes

Multi-center radiology-pathology data in 102 patients with solitary HCC ≤5 cm not amenable to ablation reported modified Response Evaluation Criteria in Solid Tumors (mRECIST) complete and partial response of 47% (47/99) and 39% (39/99), a median time to progression of 33.1 months, a median overall survival of 53.4 months, and complete pathologic necrosis in 17 of the 33 explanted tumors (52%) [21]. Long-term single-center data in 70 patients with solitary HCC ≤5 cm showed 59% complete and 90% overall imaging response (EASL) with a median overall survival of 6.7 years (approximately 80 months) after excluding transplanted patients [22]. Superselective delivery in 20 patients produced a median tumor dose of 536 Gy with no grade 3–4 hepatotoxicity [19]. In a single-center series of 60 patients with 63 tumors treated with high-specific-activity resin microspheres, complete response was achieved in 79% of tumors (50 of 63; mRECIST) at 3 months, disease control was maintained in all evaluated tumors at 6 and 12 months, and no grade 4 adverse events were recorded [23].

Comparative studies of RS with alternative locoregional therapies are shown in Table 2. Compared to trans-arterial chemoembolization (TACE) combined with microwave ablation in solitary HCC up to 3 cm, complete response rates did not differ significantly in a retrospective propensity-matched analysis (target complete response 85.0% in both arms, *p* = 0.74; overall complete response 82.5% versus 85.0%, *p* = 0.94), with no difference in time to progression (11.6 versus 11.1 months) or in mean overall survival (30.8 versus 42.7 months, *p* = 0.80; *p* > 0.99 after matching) [24]. Compared to selective chemoembolization, RS produced a higher complete response rate (81.8% vs. 49.1%) and a longer time to secondary therapy (700 vs. 246 days) [25]. Compared to percutaneous microwave ablation in solitary HCC under 4 cm, propensity-matched response rates did not differ significantly (RS 90.9% vs. MWA 82.6%, *p* = 0.548), with no significant difference in overall survival (mean 59.0 vs. 44.3 months, *p* = 0.203), but RS achieved significantly longer target-tumor progression-free survival (mean 57.8 vs. 38.6 months, *p* = 0.005) [26]. However, it is important to note that ablation margin status was not reported, so neither response nor local control could be stratified by margin, and margin was not among the matching covariates [26]. Compared to segmental chemoembolization, segmental ^90^Y significantly prolonged progression-free survival [564 vs. 271 days, propensity score (PS)-adjusted *p* = 0.002] [27]. In solitary HCC measuring ≤8 cm, tumor progression (5% versus 8%) and overall progression (33% versus 32%) were reported after radiation segmentectomy and surgical resection, respectively, and radiation segmentectomy was associated with fewer major adverse events (no events post-RS compared to 20% post-resection, *p* < 0.001). However, median time to progression was shorter with radiation segmentectomy (21.9 months versus 29.4 months, *p* = 0.03), although this difference was not observed in subgroups with fibrosis or thrombocytopenia, or in multivariate analysis (hazard ratio 1.16, *p* = 0.71). Cohorts differed in liver function, fibrosis stage, and tumor size; propensity matching was not feasible, precluding direct comparison [28].

Prospective and large-cohort data anchor the HCC evidence. The single-arm prospective RASER study treated 29 patients with solitary HCC ≤ 3 cm at a median tumor dose of 1004.6 Gy (mean 1462.6 Gy in tumors achieving complete pathologic necrosis), achieving 83% complete response and 96% two-year survival [20]. The multi-center retrospective LEGACY study of 162 patients with solitary HCC ≤8 cm reported an objective response rate of 88.3% by blinded central review and three-year overall survival of 86.6% [17]. In a 38-patient series in which treatment was prescribed to a tumor perfusion dose greater than 190 Gy (mean 349 Gy), RS converted 52% (13/25) of patients outside the Milan criteria and 47% (9/19) of those outside the University of California, San Francisco (UCSF) criteria to within the transplant criteria, and rendered 27% of initially unresectable patients anatomically resectable [29].

### 3.2. Dose–Response and Pathologic Necrosis

Explant studies provide the strongest validation of an ablative dose threshold (Table 3). In 33 patients with 37 tumors treated before transplantation, the complete pathologic necrosis rate increased from 53% at doses above 190 Gy to 75% for doses above 500 Gy [2]. A confirmatory multi-center analysis of 45 explants reported a median dose of 240 Gy with complete pathologic necrosis in 67% overall and in 100% of specimens receiving more than 400 Gy [30]. In a large explant series, a seven-center consortium of 303 patients with 364 treatment-naive tumors treated with glass microspheres before transplantation, complete pathologic necrosis was achieved in 68% of tumors overall and in 76% (184 of 241) of those treated with more than 400 Gy. Microsphere activity of 1087 Bq or greater, single-compartment dose of 440 Gy or greater, and an angiosome-to-tumor volume ratio of 16 or greater were independent predictors, and complete pathologic necrosis was achieved in 91% (57 of 63) of tumors meeting all four optimized thresholds, including particle density of 8000 microspheres/mL or greater [31]. Individually, the area under the curve was 0.67 for dose, 0.57 for microsphere activity, and 0.54 for particle density ≥ 6500/mL, compared with 0.73 when all four parameters were combined [31]. Smaller tumors were also more likely to achieve complete pathologic necrosis, both in that series (73% of tumors ≤ 3.3 cm versus 48% of larger tumors) [31] and in a voxel-dosimetry cohort [9]. Treatment intensification across 75 tumors increased CPN and identified specific activity as an independent determinant [10], and a comprehensive analysis of 61 tumors found that those achieving CPN had higher specific activity (1242 vs. 867 Bq), tumor dose (1086 vs. 738 Gy), and angiosome dose (577 vs. 352 Gy) [11]. Outcomes did not differ significantly by etiology in 142 patients with nonalcoholic fatty liver disease (NAFLD) versus hepatitis C virus (HCV)-related HCC, with complete pathologic necrosis of 63% and 54% respectively at median single-compartment doses of 508 and 452 Gy [32]. Within that cohort, among all tumors treated with ≥500 Gy, complete pathologic necrosis reached 83% when specific activity was ≥327 Bq versus 40% when specific activity was <327 Bq [32]. Biochemical safety of ablative treatment was characterized as a function of percent liver treated (median, 12.6%) at a median single-compartment dose of 403 Gy, with a percent-liver-treated threshold near 14.5% marking increased risk of hepatic deterioration (in ALBI grade or Child–Pugh class) in patients with baseline ALBI 2 or Child–Pugh B function, while no threshold was identified for ALBI 1 or Child–Pugh A patients [18].

In a resin-microsphere explant cohort, complete pathologic necrosis was associated with higher tumor absorbed dose and occurred in all tumors receiving a mean dose ≥433 Gy [33]. Notably, all tumors in which ≥4% of the volume received <100 Gy showed incomplete pathologic necrosis, despite an adequate prescribed dose. These findings suggest that ablative intent requires dose coverage of the entire tumor volume, not just an adequate mean, since even small underdosed regions can permit residual viable tumor.

Machine-learning models applied to magnetic resonance imaging (MRI) radiomics have begun to predict response to RS in HCC before it is apparent on conventional imaging. In a 76-patient single-center cohort, a model integrating baseline and early (1–2 month) post-treatment MRI predicted complete response at 4–6 months with an area under the receiver operating characteristic curve (AUC) of 0.89 on internal validation [34]. A subsequent, partially overlapping cohort from the same institution (154 patients) restricted analysis to pre-treatment MRI and found that radiomics combined with clinical parameters achieved fair discrimination (AUC: 0.74), exceeding clinical and α-fetoprotein-based models [35]. Together these findings suggest that image-based phenotyping may help identify likely responders before treatment, though both studies were single-center with overlapping cohorts and no external validation, leaving clinical readiness unproven.

**Table 3:** HCC dosimetry and pathologic necrosis.

Study	N (Tumors)	Focus	Key Dosimetric Finding
Toskich et al. [2]	33 (37)	Explant dose–response	CPN 75% > 500 Gy vs. 53% > 190 Gy
Gabr et al. [30]	45 explants	Dose-necrosis	CPN 67%; CPN in all >~400 Gy; median 240 Gy
Berman et al. [31]	303 (364)	Multi-center explant/parameters	CPN 68%; 76% > 400 Gy; SA ≥ 1087 Bq, dose ≥ 440 Gy, ATR ≥ 16 independent
Montazeri et al. [10]	75 tumors	Intensification/SA	Intensification ↑ CPN; SA ≥ 327 Bq independent
Montazeri et al. [11]	61 tumors	Comprehensive CPN	CPN: SA 1242 vs. 867 Bq; tumor 1086 vs. 738 Gy
De la Garza-Ramos et al. [32]	142	NAFLD vs. HCV	CPN 63%/54% by etiology (*p* = 0.46); 83% vs. 40% at ≥500 Gy + SA ≥ 327 Bq
De la Garza-Ramos et al. [18]	141	Biochemical safety	Median dose 403 Gy; toxicity rises at %LT ≥ 14.5%
Orcajo Rincón et al. [5]	24	Voxel dosimetry	Max tumor-absorbed dose predicts response
Sandow et al. [9]	56	Voxel/sphere conc.	Sphere conc. and D70/90/99 vs. outcome
Villalobos et al. [4]	81 (61G/20R)	Glass vs. resin	Distinct device dose/SA thresholds
Marinelli et al. [34]	76	ML MR radiomics	Predicts early response
Stocker et al. [35]	154	ML MRI	Pre-treatment prediction of response

HCC = hepatocellular carcinoma; N = number; G = glass; R = resin; CPN = complete pathologic necrosis; SA = specific activity; Bq = becquerel; Gy = gray; D70/D90/D99 = minimum absorbed dose to 70%/90%/99% of tumor volume; %LT = percent liver treated; ML = machine learning; MR/MRI = magnetic resonance imaging; NAFLD = nonalcoholic fatty liver disease; HCV = hepatitis C virus.

HCC is hypervascular and has a predominantly arterial blood supply, so arterially delivered microspheres are taken up preferentially by tumors relative to the background parenchyma [35]. This preferential tumor uptake is what makes tumor-volume dosimetry meaningful in HCC, and it is the basis on which explant studies tie higher tumor-directed absorbed dose to greater pathologic necrosis. The practical objective is therefore to maximize tumor-absorbed dose through superselective catheter positioning, with reported ablative targets of roughly 400–500 Gy for glass [2,17], a lower tumor-dose threshold near 300 Gy for resin [3], and adequate specific activity [10]. The degree of selectivity may also matter: in a multi-center explant series, an angiosome-to-tumor volume ratio of 16 or greater independently predicted complete pathologic necrosis, and superselective administration was noted to lower this ratio [31].

## 4. Colorectal Liver Metastases

The dedicated RS evidence in colorectal liver metastases is limited (Table 4). A 10-year single-center experience in 45 heavily pretreated patients with liver-dominant metastatic colorectal cancer used escalated segmental and modified-lobar dosing, reporting 98% local disease control and a median overall survival of 41.9 months, with extrahepatic disease and a high neutrophil-to-lymphocyte ratio as adverse prognostic factors [36]. A single-center dosimetric analysis of 36 patients with 57 metastases treated above 190 Gy found that a mean tumor dose ≥400 Gy combined with a 5 mm margin mean absorbed dose ≥350 Gy was associated with 83.3% one-year local tumor progression-free survival; median overall survival was 14.3 months, consistent with the systemic disease burden [37].

Colorectal metastases are typically hypovascular with central necrosis and viable tumor concentrated peripherally, which makes it plausible that the dose delivered to the peritumoral rim, rather than the mean dose attributed to the whole tumor volume, governs local control [38,39]. The margin-dose association reported by the single dosimetric study above is consistent with this idea [37], and voxel-based dosimetry methods developed in HCC provide the tools to test it [5,9]. This hypothesis, primarily based on a single retrospective study, necessitates prospective validation through post-treatment dose mapping and pathological correlation [40,41].

## 5. Intrahepatic Cholangiocarcinoma

A 16-patient glass-microsphere series delivering ≥190 Gy reported a median overall survival of 7 months following the procedure, with significantly longer post-treatment survival among patients who had not received chemotherapy before radioembolization (26.8 vs. 5.9 months, *p* = 0.03), and no 30-day mortality [42]. Dose escalation above 400 Gy in 10 patients with 13 tumors (1.5–13.6 cm) achieved 100% objective response, suggesting that RS may be applicable to tumors beyond thermal-ablation size limits, although this requires further validation [43]. In 15 patients with unresectable early-stage intrahepatic cholangiocarcinoma (iCCA), RS and modified radiation lobectomy at a median dose of 308 Gy were safe and effective (Table 5) [44].

iCCA shows heterogeneous and inconsistent tumoral vascularity, being hypovascular with peripheral rim enhancement in many cases, but hypervascular in others [45], so no single tumor-dose threshold has been established, and the optimal dosimetric approach requires further validation.

**Table 5:** Intrahepatic cholangiocarcinoma.

Study	N (Tumors)	Design	Dose	Key Outcome
Kumar et al. [42]	16	Single-center retrospective	Median 225.1 Gy	OS 22.7 from diagnosis; no prior chemotherapy (*n* = 6) vs. prior chemotherapy (*n* = 10): 26.8 vs. 5.9 (*p* = 0.03)
Yu et al. [43]	10 (13)	Single-center retrospective	Glass >400 Gy	Objective response 100%; up to 13.6 cm
Gupta et al. [44]	15	Single-center retrospective	Median 308 Gy	Safe/effective, early-stage iCCA

iCCA = intrahepatic cholangiocarcinoma; OS = overall survival; Gy = gray.

## 6. Neuroendocrine and Mixed Metastatic Disease

A cohort of 18 patients with 23 neuroendocrine liver metastases across tumor grades I-III reported an objective response of 83% by RECIST 1.1 in all 23 tumors and 100% by mRECIST in the 12 hypervascular tumors assessed by enhancement criteria, and a median overall survival of 69.4 months, with time to progression in the treated tumor not reached over a median follow-up of 31.9 months [46]. Hypervascular tumors, or a liver tumor burden below 25%, were associated with significantly improved overall survival, consistent with the vascular-uptake rationale for radiation segmentectomy [46]. Neuroendocrine tumor (NET)-specific dose thresholds and pathologic correlation have not yet been established.

Cross-histology series suggest feasibility (Table 6), although applicability across histologies requires further validation. In a multi-institutional study of 36 patients with 53 tumors of mixed origin, the authors reported a 92% disease control rate with no high-grade hepatic toxicity [47]. In limited metastatic liver disease treated above 190 Gy, a 2-year local tumor control rate of 83% was reported [16]. In a cohort of 16 patients with oligometastatic secondary malignancies, RS was safe and effective, with grade 3 toxicity in two patients (13.3%) [48]. Similarly, in a 10-patient series (7 colorectal), disease control was documented in 7 of 10 patients with only a single, self-limited grade 1 adverse event, supporting the safety of segmental radioembolization for hepatic metastases [49].

## 7. Heterogeneity and the Limits of Cross-Study Comparison

Meaningful cross-study synthesis is constrained by heterogeneity that operates at several levels of the evidence, some of which reflect genuine variation in technique and some of which render the reported outcomes non-comparable in principle. Two of these differences are categorical rather than continuous and warrant emphasis because they preclude direct numerical comparison. First, the microsphere device differs across series: glass and resin microspheres carry markedly different specific activities and particle numbers and are prescribed using different dosimetric conventions, such that a nominal absorbed dose in one system does not correspond to the same physical or biological quantity in the other [4]. Second, and more fundamentally, the dosimetric method itself is not standardized. Reported doses are variously derived from single-compartment MIRD, partition, or post-treatment voxel-based models, which estimate distinct quantities; a tumor dose reported as a single-compartment perfused-volume value, a partition-based compartmental value, and a voxel-based D50 are not interconvertible, so apparent agreement or disagreement between thresholds may be an artifact of method rather than of biology. Tumor absorbed dose, correlated with pathologic and imaging evidence of tumor destruction, could serve as the common denominator, with histology-specific thresholds.

Superimposed on these categorical differences is continuous heterogeneity in dose and in patient selection. Doses described as ablative span a wide range, from a single-compartment threshold of 190 Gy [21] to tumor doses reaching 1462.6 Gy (759.9 to 2165.3) in tumors achieving complete pathologic necrosis in a prospective early-stage cohort [20], and device-specific thresholds diverge between glass and resin [3,4]. Selection heterogeneity extends well beyond tumor size to encompass tumor number, Barcelona Clinic Liver Cancer (BCLC) stage, and hepatic functional reserve: series have variously restricted enrollment to solitary hepatocellular carcinoma with diameter up to 5 cm with Child–Pugh A function [22] or to very-early and early-stage (BCLC 0 to A) disease [20], whereas earlier cohorts extended across BCLC stages A through C [1]. Because these series differ in case mix across several of the predefined inclusion criteria, apparent survival differences between them, for example 26.9 versus 53.4 months in hepatocellular carcinoma, cannot be attributed only to the treatment and may substantially reflect selection as a confounder of treatment effect and underlying disease biology [1,21]. Assessing response compounds the issue. A tumor that loses vascularity without a reduction in size is classified as a complete response by mRECIST but as stable disease by RECIST 1.1, rendering response rates incomparable across studies employing different criteria. The extent of this discrepancy is significant: in a radiation segmentectomy cohort of 38 patients evaluated under both criteria, the objective response was 84.2% by mRECIST compared to 31.6% by RECIST 1.1 (complete response 31.6% versus 2.6%, stable disease 10.5% versus 63.2%), while progressive disease (5.3%) and disease control (94.7%) remained consistent. The mean size reduction was 7.6% in lesions classified as stable by RECIST 1.1, compared to a 76.5% reduction in enhancing diameter in the lesions classified as responders by mRECIST. This probably indicates that the discrepancy arises from the reclassification of devascularized tumors without size change, rather than from any difference in disease control [29].

The choice of response criterion may therefore depend, at least in part, on tumor vascularity. mRECIST, validated in hepatocellular carcinoma [47], may be considered for hypervascular metastases, which are indistinguishable from hepatocellular carcinoma on dynamic imaging [39]. In hypovascular disease, its applicability is less certain: intrahepatic cholangiocarcinoma and colorectal metastases often show heterogeneous or predominantly peripheral enhancement, which may limit enhancement-based assessment. Several series in these tumors have therefore used RECIST 1.1 [44,45], and attenuation-based and metabolic criteria have been proposed as alternatives [50].

Timing constrains all criteria, and this constraint is specific to segmental delivery. Early size increase reflects peritumoral edema, hemorrhage, and hyperenhancement persisting up to three months, along with a transient increase in treated tumor size in responding patients; in that cohort, the mRECIST category changed between the 1.7-month and 4.5-month scans in 30 of 76 patients, and 28% of those initially scored as having viable tumors ultimately achieved complete response [34]. Loss of T1 signal confined to the treated angiosome has been proposed as the segmentectomy equivalent of an ablation margin [2].

Eligibility and contraindication thresholds are likewise unstandardized. The permissible proportion of liver treated, defined as the volume of the irradiated territory relative to total liver volume, together with the acceptable degree of hepatic dysfunction and the handling of portal vein tumor thrombosis, differs across series. Treating more than approximately 14.5% of the liver volume has been associated with biochemical toxicity, but only in patients with compromised baseline hepatic function (albumin-bilirubin grade 2 or Child–Pugh B), indicating that even this threshold is conditional on hepatic reserve rather than fixed [18]. Current consensus frameworks nonetheless articulate largely relative rather than absolute contraindications [12]. Because eligibility sits upstream of every reported outcome, this variability is not a peripheral issue, but a primary source of the between-study divergence tabulated above.

## 8. The Unresolved Segmental Dose Prescription

Radiation segmentectomy is prescribed and delivered as an absorbed dose to the targeted hepatic segment or perfused volume, whereas oncologic local control is governed by the dose delivered to the tumor and, in hypovascular disease, potentially to the peritumoral margin, a hypothesis not yet validated prospectively [37]. These are not equivalent: paired dosimetric reports demonstrate that the tumor dose substantially exceeds the segmental dose. In a prospective early-stage cohort, tumors achieving complete pathologic necrosis received a mean perfused-segment dose of 776.5 Gy (532.4 to 1020.6) versus a mean tumor dose of 1462.6 Gy (759.9 to 2165.3), a difference the investigators attributed to preferential arterial blood flow to the tumor [20]; comparable relationships are reported in the original single-center series (median segmental dose 521 Gy versus tumor dose 1214 Gy) [1] and in explant analyses of complete pathologic necrosis (median angiosome dose 577 Gy versus tumor dose 1086 Gy) [11]. The clinically operative question is therefore not only what tumor and margin doses are required for durable local control, but what segmental, perfused-volume dose must be prescribed to achieve them. This segment-to-tumor dose relationship is not fixed; it depends on the fraction of the perfused volume occupied by tumor and, critically, on tumor vascularity and primary histology, which govern the preferential concentration of microspheres within the tumor relative to the background parenchyma [9,44]. No prospective study has defined the segmental prescription required to reliably achieve a target tumor dose across tumor phenotypes, and the existing dose–response literature reports tumor or perfused-volume doses in isolation rather than the prescription relationship that links them.

This relationship is unlikely to be a single constant. It should vary with the ratio of tumor to background-parenchymal arterial supply, which governs how preferentially microspheres deposit in tumor. The microsphere device matters as well, since glass and resin differ in specific activity and particle number, and therefore in how a given segmental dose is deposited. Finally, the number of infusion sites across which the segmental dose is delivered influences how uniformly the dose covers the tumor. The calibration date used to decay-correct Y-90 activity is a further, frequently understated source of dosimetric error, given the isotope’s short half-life, where even small timing discrepancies translate into clinically meaningful differences in delivered activity. Defining the segmental prescription that reliably achieves a target tumor and margin dose will therefore require characterizing this relationship as a function of tumor phenotype and delivery technique, rather than assuming a fixed segment-to-tumor conversion.

Numerous studies referenced in this review did not limit themselves to segmental delivery. In the LEGACY study, selective infusion was administered to 155 out of 162 patients (95.7%), with lobar infusion in 3 patients (1.9%) and mixed infusion in 4 patients (2.5%). The treatment approach was categorized as radiation segmentectomy in 95 patients (58.6%) and radiation lobectomy in 4 patients (2.5%) [17]. In the non-HCC series, modified radiation lobectomy was conducted in 2 out of 45 patients with colorectal liver metastases [36]. For intrahepatic cholangiocarcinoma, 6 out of 16 patients underwent radiation lobectomy [42], while lobar treatment in addition to radiation segmentectomy was reported in 6 out of 10 patients [43]. Furthermore, modified radiation lobectomy, including lobar infusion in 2 patients, was performed in 3 out of 15 patients [44]. Additionally, the DOSISPHERE-01 trial employed a lobar approach [8]. The intrahepatic cholangiocarcinoma cohort, cited for tumor vascularity, received treatment through whole-liver (54%), single-lobe (43%), or superselective (3%) radioembolization [45]. The international working-group recommendations encompass both radiation segmentectomy and lobar treatment [12].

## 9. Future Perspectives and Conclusions

Several priorities follow from this synthesis. Histology-specific dosimetry should be validated prospectively; in particular, the colorectal margin-dose hypothesis warrants testing with post-treatment voxel dose mapping and pathologic correlation rather than adoption as standard on the basis of a single retrospective analysis [37]. Post-Y90 tissue sampling for direct correlation of tissue radiation exposure to pathologic changes may offer important objective information to inform dosimetry thresholds to achieve tumor cell death [40,41]. Beyond validating individual thresholds, the field should define the segmental-to-tumor dose relationship as a function of tumor vascularity and primary histology. Multicompartment, voxel-based dosimetry could potentially be advanced as the default standard for both planning and post-treatment verification. Standardization of eligibility criteria and absolute contraindications is equally necessary, since inconsistent selection is upstream of the outcome heterogeneity that currently limits synthesis. Prospective investigation should be expanded in non-HCC disease using the single-arm design that proved feasible in hepatocellular carcinoma [20], and the direct comparison of radiation segmentectomy with surgical resection, repeatedly identified as an evidence gap, should be pursued. Finally, image-based response prediction, where machine-learning radiomics has shown early promise, merits prospective validation as a tool for pre-treatment patient selection and early response prediction [34,35]. Collectively, these priorities converge on a single objective: to replace the current patchwork of device-specific, method-specific, and histology-agnostic practice with a standardized, biologically informed dosimetric framework.

Biological mechanisms of radioresistance may also be relevant to residual disease. In radiotherapy, surviving tumor cells can persist in plastic, therapy-tolerant states, stabilized by hypoxic, vascular, stromal, metabolic, and immune microenvironments [51]. Post-radiotherapy recurrence is frequently enriched in hypoxic regions, perivascular zones, or invasive fronts, and it has been proposed that a human spatial atlas of radiotherapy response should integrate tissue measurements with delivered radiation dose, recurrence site, and clinical outcome [51]. The relevance of these observations to ^90^Y radioembolization remains to be established. With regard to immune responses following ^90^Y radioembolization, tumors resected from patients with hepatocellular carcinoma after treatment exhibited greater infiltration of activated CD8+ T cells, natural killer cells, and natural killer T cells, along with fewer regulatory T cells, compared with treatment-naïve tumors [52]. Additionally, systemic immune activation was observed in the peripheral blood of patients with a sustained response [52]. These sustained responders also demonstrated higher percentages of PD-1+ and Tim-3+ CD8+ T cells in peripheral blood than non-responders and transient responders, both before and three months after treatment [52]. A prediction model based on pretreatment peripheral blood immune profiles predicted sustained response with an accuracy of 75.0% in an independent test cohort of eight patients [52]. Based on these findings, it has been proposed that ^90^Y radioembolization followed by immune checkpoint inhibition of the PD-1/PD-L1 or Tim-3 pathways, particularly in sustained responders, may enhance the clinical response [52].

This review is subject to several limitations. Firstly, it is a narrative review rather than a systematic one; although the search strategy and selection criteria are detailed, no formal meta-analysis was conducted. Secondly, the evidence primarily originates from retrospective, often single-center studies with small patient cohorts, which restricts statistical power and generalizability. Thirdly, selection bias is probable, as radiation segmentectomy was frequently administered to patients deemed unsuitable for surgery or ablation, and explant studies include only those who subsequently underwent transplantation or resection. Fourthly, the studies included varied in patient demographics and characteristics, such as tumor size, stage, underlying liver disease, and prior treatment, as well as in microsphere type and dosimetry methods, which complicates cross-study comparisons. Fifthly, several cohorts from the same institutions were reported in multiple publications, making patient overlap across studies a possibility. Sixthly, some series combined segmental and lobar treatment, and some evidence cited for context is derived from non-segmental radioembolization. Seventhly, comparisons with ablation, resection, and chemoembolization are retrospective, and the lack of statistically significant differences in these studies does not confirm clinical equivalence. Lastly, the safety of omitting MAA, the role of peritumoral margin dosimetry, and the application of radiation segmentectomy to non-HCC indications are supported by limited data and necessitate further validation.

Radiation segmentectomy represents a potentially curative treatment for certain patients with early-stage HCC, as evidenced by explant dose–response data and supported by prospective studies and large cohort analyses. However, comparisons with ablation and resection are based on retrospective studies, and the lack of statistically significant differences in these studies does not confirm clinical equivalence. In non-HCC tumors, the evidence supports feasibility and durable responses while remaining largely retrospective, thus requiring further validation. The unifying principle is that vascular biology should determine dosimetric strategy; where that principle is supported by pathologic correlation, as in HCC, it is ready for practice, and where it is still inferred, as in colorectal metastases, it should drive the next prospective studies rather than premature standardization.

## Figures and Tables

**Table 1:** Segmental (perfused) versus tumor absorbed dose, where both are reported.

Study	Segmental/Perfused Dose	Tumor Absorbed Dose
Riaz et al. [1]	521 Gy (range 67–2158)	1214 Gy (range 61–9934)
Padia et al. [19]	254 Gy (range 105–1055)	536 Gy (range 203–1618)
Kim et al. [20]	584 Gy (range 181–3340). CPN Subgroup: 776.5 (532.4–1020.6)	1004.6 Gy (range 190.8–3730). CPN Subgroup means 1462.6 (759.9–2165.3)
Montazeri et al. [11]	480 Gy (IQR 306–679). CPN Subgroup: 577 (IQR 406–735)	1042 Gy (IQR 668–1496). CPN Subgroup: 1086 (IQR 848–1632)
Sandow et al. [9]	474 Gy (range 177–1290)	732 Gy (range 252–1776); D99: 463 Gy (range 150–2035)

IQR = interquartile range; CPN = complete pathologic necrosis; D99 = minimum absorbed dose to 99% of tumor volume; Gy = gray.

**Table 2:** HCC clinical outcomes.

Study	N	Design	Sphere	Response	TTP/OS
Riaz et al. [1]	84	Single-center retrospective	Glass	59% IR; 81% necrosis	TTP 13.6; OS 26.9
Vouche et al. [21]	102	Multi-center retrospective	Glass	CR 47%/PR 39%	TTP 33.1; OS 53.4
Padia et al. [19]	20	Single-center retrospective	Glass	High response; no grade 3–4 toxicity	-
Biederman et al. [24]	41	Retrospective, PSM RS vs. TACE + MWA	Glass	Target CR: 85.4% (35/41) unmatched, 85.0% (34/40) matched	11.1 months (95% CI 8.8–25.6) unmatched, 11.6 months (95% CI 7.7–17.3); OS 30.8 *
Biederman et al. [25]	55	Retrospective	Glass	CR 81.8% vs. 49.1%	TTST 700 vs. 246 days
Lewandowski et al. [22]	70	Single- center retrospective	Glass	CR 59%; OR 90%	OS 6.7 yr (LT excl.)
Arndt et al. [26]	34	Retrospective, PSM RS vs. MWA	Glass	CR + PR 90.9%	mean target-lesion PFS 57.8; OS 59.0 No significant difference in overall survival (mean 59.0 vs. 44.3 months, *p* = 0.203)
De la Garza-Ramos et al. [28]	57	Retrospective RS vs. resection	Glass	CR 87%/PR 11% (47 patients at 6 months); 0 major AE (57 patients)	TTP 21.9
Kim et al. [20]	29	Prospective single-arm	Glass	CR 83%; 96% 2-yr survival	-
Sarwar et al. [3]	67	Single-center retrospective	Resin	ORR at 6 months per-lesion 94%	OS 26
Villalobos et al. [23]	60	Single-center retrospective	Resin	CR at 3 months per-tumor 79% (mRECIST)	-
Son et al. [29]	38	Retrospective	Glass	Conversion to LT/resection	-

* mean value; otherwise medians. N = number of patients; AE = Adverse events; retro = retrospective; PSM = propensity score matching; MWA = microwave ablation; TACE = trans-arterial chemoembolization; CR = complete response; PR = partial response; IR = imaging response; ORR = objective response rate; TTP = time to progression; OS = overall survival; TTST = time to secondary therapy; LT = liver transplant.

**Table 4:** Colorectal liver metastases.

Study	N (Tumors)	Design	Key Finding	OS (mo)
Gordon et al. [36]	45	Single-center retrospective, escalated dose	98% local control; NLR and EHD prognostic factors	41.9
Dimopoulos et al. [37]	36 (57)	Single-center retrospective, voxel dosimetry	MTD ≥ 400 + 5 mm MMAD ≥ 350 Gy → 83.3% 1-yr LTPFS	14.3

MTD = mean tumor dose; MMAD = margin mean absorbed dose; NLR = neutrophil-to-lymphocyte ratio; EHD = extrahepatic disease; LTPFS = local tumor progression-free survival; OS = overall survival;; Gy = gray.

**Table 6:** Neuroendocrine and mixed/other metastases treated with radiation segmentectomy.

Study	N (Tumors)	Median Diameter, cm (Range/CI)	Primary Tumor Composition	Key Outcome
Gordon et al. [46]	18 (23)	3.7 (1.5–10.1)	Neuroendocrine, grade I–III	ORR 100% by mRECIST (83% by RECIST); median OS 69.4 mo; treated-tumor TTP not reached; improved OS with hypervascular tumors; Grade 3 lymphopenia: 3 patients (17%) Grade 3 fatigue: 1 patient (6%)
Padia et al. [47]	36 (53)	3.5 (1.2–7.3)	Colorectal 11, NET 10, sarcoma 7, other 8 (uveal melanoma 2, GIST 2, adrenocortical 1, pancreatic 1, osseous hemangiopericytoma 1, thyroid 1)	Disease control 92%; ORR 100% (mRECIST); OS 96%/83% at 6/12 mo; No grade 3–4 hepatic toxicity
Kurilova et al. [16]	10 (14)	3.0 (1.4–5.6)	Colorectal 2, breast 1, ovarian 1, NSCLC 1, pancreatic 1, gallbladder 1, gastric 1, ampullary 1, GE junction 1	2-yr local tumor control 83% at >190 Gy in limited metastatic liver disease
Chiu et al. [48]	16	3.1 (95% CI 2.3–3.9)	Colorectal 4, head and neck SCC 3, breast 2, pancreatic 1, prostatic 1, spindle cell sarcoma 1, leiomyosarcoma 1, GIST 1, NET 1, urothelial 1	Disease control 93.3% (14/15); median OS 60.9 mo; grade 3 toxicity in 2 of 15 (13.3%)
Meiers et al. [49]	10	Not reported	Colorectal 7 (colon 4, colorectal 2, rectal 1), breast 1, leiomyosarcoma 1, carcinoid 1	Pilot safety/feasibility series; Disease control in 7/10; mean PFS 7.1 mo; Grade 1 toxicity (self-limited hiccups due to phrenic irritation): 1/10

Median diameter is the study-reported median index tumor diameter with range in parentheses, except Chiu et al., which reported a 95% confidence interval (CI) rather than a range; Meiers et al. did not report tumor diameter. CI = confidence interval; GE = gastroesophageal; GIST = gastrointestinal stromal tumor; Gy = gray; mRECIST = modified Response Evaluation Criteria in Solid Tumors; N = number of patients; NET = neuroendocrine tumor; NSCLC = non-small cell lung cancer; ORR = objective response rate; OS = overall survival; RECIST = Response Evaluation Criteria in Solid Tumors; SCC = squamous cell carcinoma; TTP = time to progression.

## Data Availability

No new data were created or analyzed in this study. Data sharing is not applicable to this article.
